# Berberine is sufficient to restore the destroyed seminiferous tubule structure and hypospermatogenesis in diabetes mellitus

**DOI:** 10.1002/ctm2.193

**Published:** 2020-10-11

**Authors:** Xintao Gao, Zhuo Liu, Jingyu Song, Yucong Zhang, Hongyang Jiang, Delin Ma, Jiaxin Wang, Penghui Yuan, Rui Li, Jian Bai, Tao Wang, Shaogang Wang, Jihong Liu, Xiaming Liu

**Affiliations:** ^1^ Department of Urology Tongji Hospital Tongji Medical College Huazhong University of Science and Technology Wuhan Hubei P. R. China; ^2^ Department of Geriatrics Tongji Hospital Tongji Medical College Huazhong University of Science and Technology Wuhan Hubei P. R. China; ^3^ Department of Endocrinology Tongji Hospital Tongji Medical College Huazhong University of Science and Technology Wuhan Hubei P. R. China; ^4^ Department of Reproductive Medicine Center Tongji Hospital Tongji Medical College Huazhong University of Science and Technology Wuhan Hubei P. R. China

Dear Editor,

Due to the lower‐age tendency of diabetes incidence, male fertility is threatened by diabetes mellitus.^1^, ^2^ Our team unprecedentedly extended the untreated diabetes duration of diabetes from 1 week to 12 weeks to damage testes, which provided a strong basis for the conclusion that berberine (BB) could significantly repair the damaged tight junctions in diabetic rats and play a potential role in rescuing diabetic hypospermatogenesis via inhibiting JAK2/MAPK pathway.

After the adaption for a month, 60 six‐week age male wild‐type Sprague–Dawley rats were used to build a type 1 diabetes rat model, and the induction process was based on previous study.^3^ After a 12‐week duration of diabetes, intragastric administration was used for a 4‐week treatment of BB (Figure 1). And rats were randomly divided into five groups: normal control, diabetes mellitus (DM) rats control, BB‐treated DM rats with three doses. In vitro, the cell culture medium with high concentration of D‐glucose was used to simulate hyperglycemia prior to BB treatment.

**FIGURE 1:**
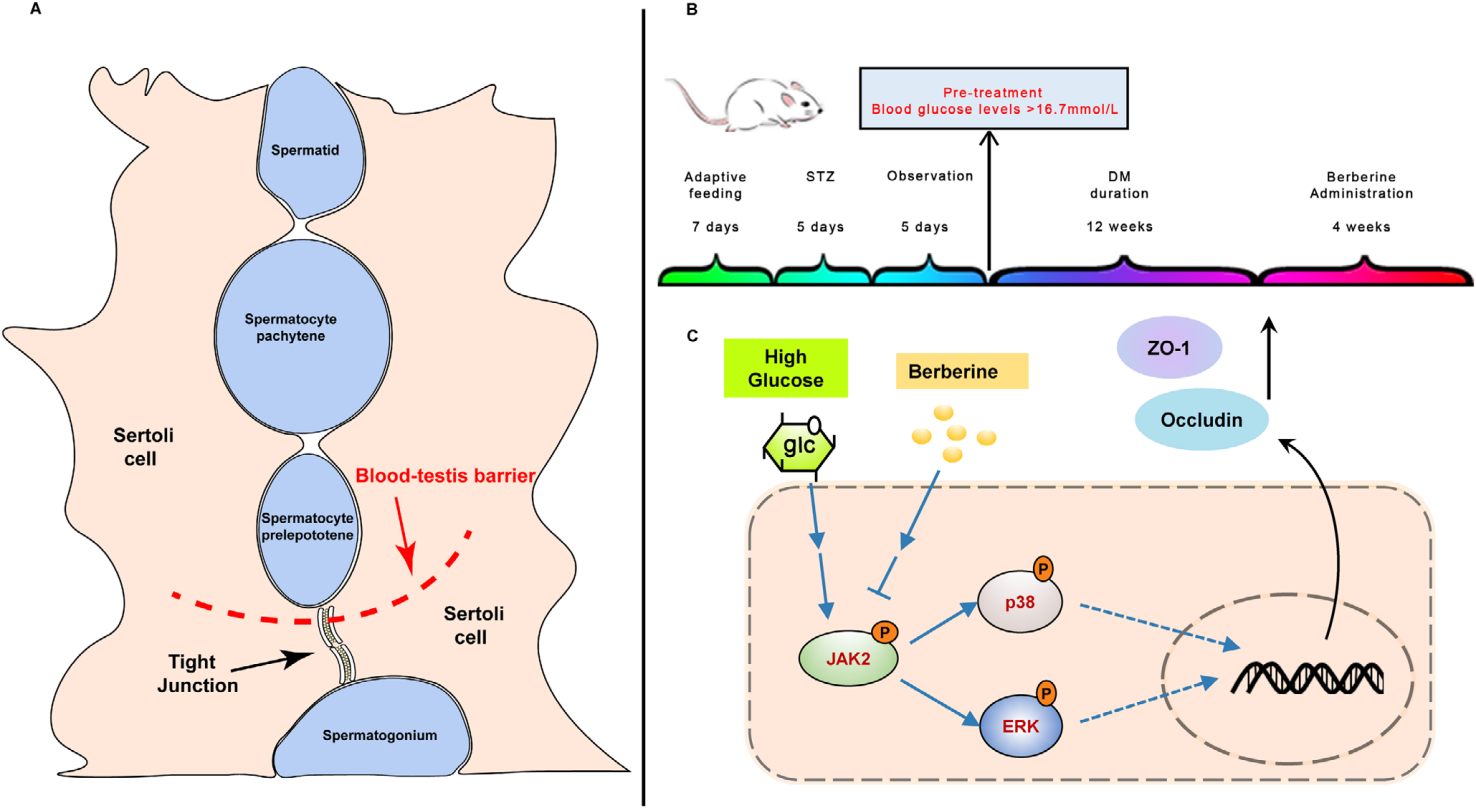
Graphical abstract. A, The positional relationship between Sertoli cells and spermatogenic cells and tight junction structure in the testis. B, Animal experiment process. C, Schematic diagram of berberine inhibiting the JAK2/MAPK pathway in Sertoli cells

The metabolic and physiological data of rats was evaluated firstly. The weight of diabetic rats decreases dramatically, while BB treatment could improve the nutritional status, but did not reduce the blood glucose levels (Supporting information Table S1). As for the condition of testes and sperm quality, the testis weight, total number of the sperms, proportion of the sperms with normal morphology and motility of the sperms kept rising upon the treatment of increasing doses of BB in the diabetic rats (Figure 2). The H&E staining results of testes further demonstrated that there was serious damage to the structures of seminiferous tubules after the 12‐week duration of diabetes (Figure 2C‐2, C‐8). The number of spermatogenic cells at different stages was dramatically reduced, and a break in tight junctions formed of Sertoli cells was also observed. However, these changes were reversed after being treated with BB for 4 weeks. The number of spermatogenic cells at different stages gradually increased, and the structure of seminiferous tubules tended to be more complete with a small amount of sperms visible in the lumen (Figure 2). By using transmission electron microscopy (TEM), we observed that the dense line in the testis of diabetic rats was broken up into sections and seemed to be very blurred, which meant a serious breakdown of the tight junctions (Figure 2D‐8, D‐9). After administration of BB, the tight junction structure recovered to varying degrees, and the Sertoli cells damage gradually became much lighter maintaining the integrity of the tight junctions (Figure 2D).

**FIGURE 2:**
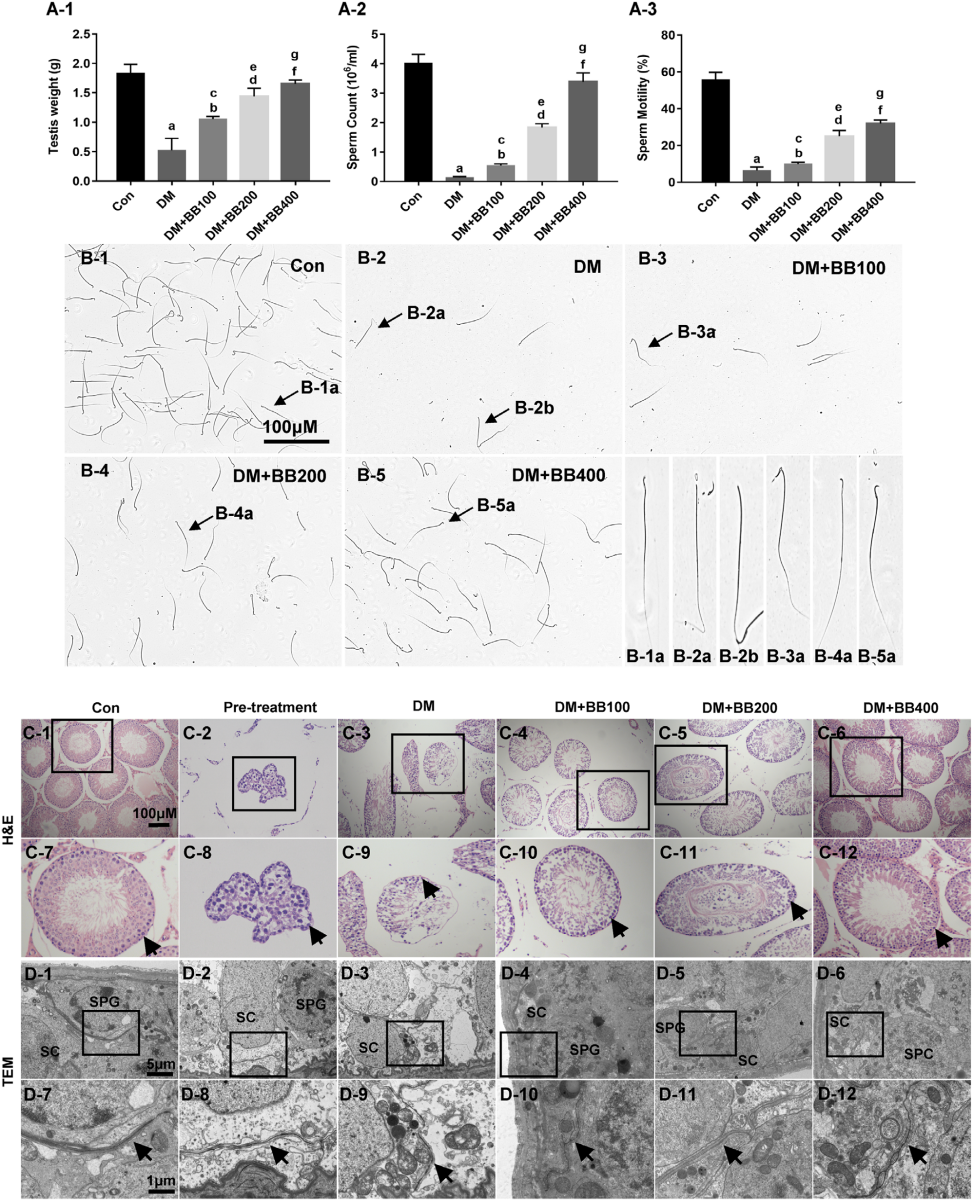
Observation and functional analyses in testes of long‐term diabetic rats. A, Testis weight and sperm quality shown as bar graphs in each group after 4‐week berberine treatment. B, Sperm morphology (× 400) in each group. C, Testicular histological analysis in rats (H&E stain, × 100). Arrows: Sertoli cells. D, The structure of testes observed by transmission electron microscope (TEM) including tight junctions and spermatogenic cells. Arrows: tight junctions. SC, Sertoli cells; SPG, spermatogonium; SPC, spermatocyte. Data are expressed as mean ± standard deviation. ^a, b,d,f^ *P* < .05 versus Con group.^c,e,f^ *P* < .05 compared with the DM group. Con, control; Pretreatment, diabetic rats before Berberine treatment; DM, diabetes mellitus; BB100, Berberine treat 100 mg/kg/d; BB200: Berberine treat 200 mg/kg/d; BB400: Berberine treat 400 mg/kg/d

Next, the expression and distribution of tight junction proteins (occludin, ZO‐1) in rat testes and Sertoli cell lines were both detected. Combining immunofluorescence results, we found that the tight junction proteins were localized to the apicolateral Sertoli cells plasma membrane and cell junctions (Figure 3A‐B, E‐F). For BB, there was a favorable trend for the expression level of tight junction proteins, similar to the morphology results (Figure 3C‐D, G‐H).

**FIGURE 3:**
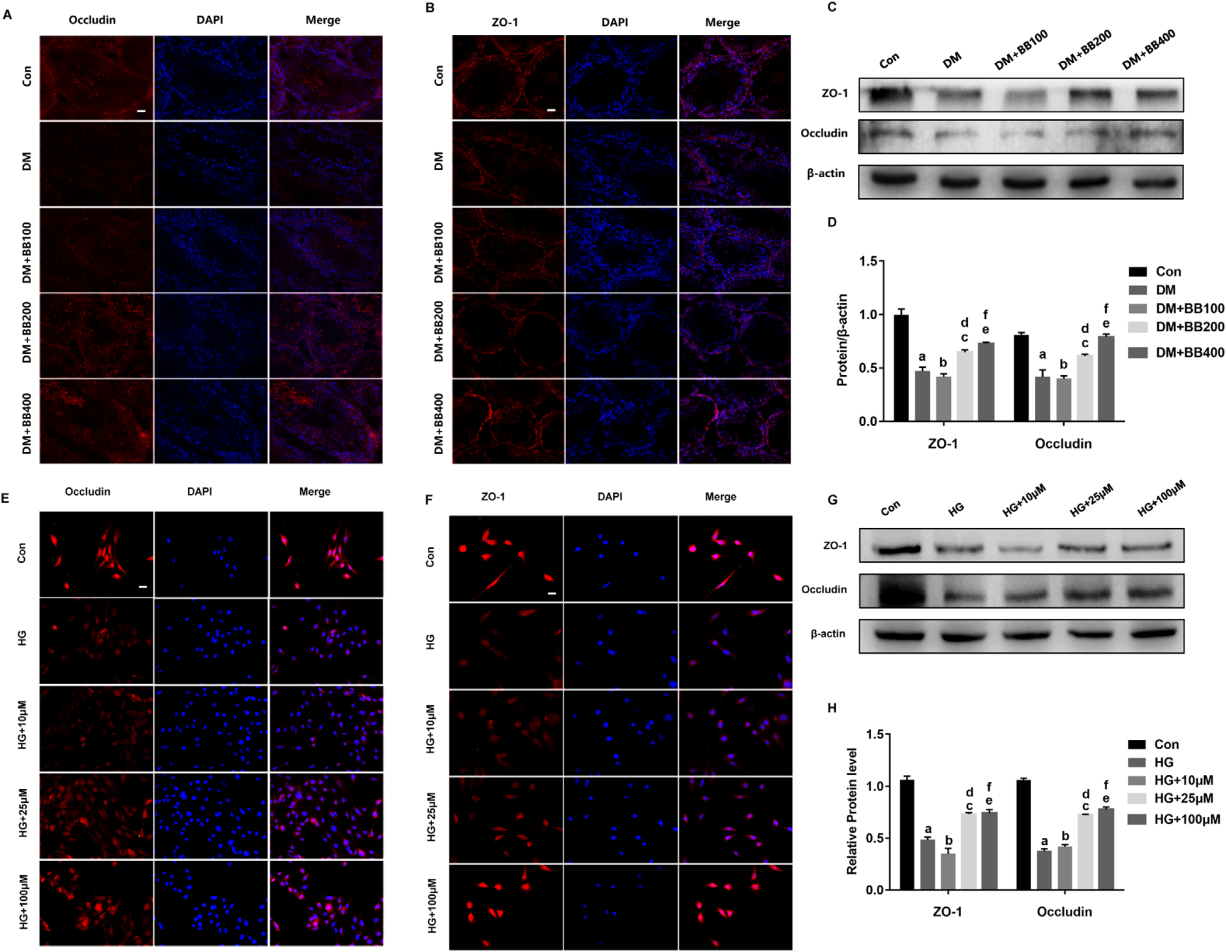
Berberine recovered the expression of tight junction proteins in Sertoli cells upon diabetic conditions in vitro and in vivo. A‐B, Immunofluorescence results of occludin and ZO‐1 in each group of testes demonstrating the increase expression in berberine treatment groups (× 400). Scale bars = 10 µm. n  =  6 for each group. C‐D, Representative Western blot results and corresponding bar graphs for ZO‐1, occludin in the testes of rats from all five group. E‐F, Immunofluorescence results of occludin and ZO‐1 in the TM4 cells of all five groups (× 400). G‐H, Representative Western blot results and corresponding bar graphs for ZO‐1, occludin in the TM4 cells of all five groups. Data are expressed as mean ± standard deviation. ^a,b,c,e^ *P *< .05 compared with the Con group. ^d,f^ *P* < .05 compared with the DM group or HG group. Con, control; DM, diabetes mellitus; BB100, Berberine treat 100 mg/kg/d; BB200, Berberine treat 200 mg/kg/d; BB400, Berberine treat 400 mg/kg/d. HG, high glucose medium (50 mM); 10, 25,100 µM: Berberine concentrations

As the MAPK pathway was reported to be involved in the regulation of tight junctions, we detected the expression and activation of p38 and ERK in Sertoli cells, which were the most important subfamily of MAPK currently known.^4^, ^5^ P38 and ERK pathways were indeed activated by phosphorylation, and the activity of these pathways was significantly inhibited by BB (Supporting information Figure S1A‐C). The recent literatures and our previous studies revealed that JAK2 played an important role in important organ damage induced by diabetes, so we speculated that the levels of JAK2 and p‐JAK2 in Sertoli cells impaired by diabetes might change. Both in vivo and in vitro, the addition of BB not only downregulated the overexpression of JAK2, but also inhibited the activity of JAK2, manifesting as a decrease of p‐JAK2 (Supporting information Figure S1D‐H). Therefore, we hypothesized that diabetes might damage tight junctions formed by Sertoli cells via activating JAK2/MAPK signaling pathway.

By exposure to a small molecular inhibitor AG490 and siRNA, we confirmed the regulatory effect of JAK2 on MAPK pathways and tight junction proteins (Supporting information Figure S2). On the other hand, we found that the inhibition of p38 and ERK signal pathways improved the expression of tight junction protein induced by high glucose (Supporting information Figure S3).

Some drugs have been found to be used to treat the tight junctions and blood‐testis barrier (BTB) damage caused by diabetes including betaine, salidroside, *Dioscorea zingiberensis* ethanol extract.^6^, ^7^, ^8^ However, the administration of all these experiments began within 7 days after injection of STZ, which was designed to check the protective effect rather than the repairing effect of the drugs. Thus, BB was the first medication being experimentally tested to have an incredible repairing effect on the diabetes related testis severe damage. In addition, the repairing effect of BB was confirmed both in vivo and in vitro with gradient concentrations. Therefore, the results demonstrated that BB was sufficient to repair the damaged testicular seminiferous tubules in long‐term diabetes by reconstructing tight junctions and BTB.

In conclusion, BB demonstrated a very powerful repairing effect on the diabetes related testis damage. This repairing effect is much more meaningful in the real clinical situation, which indicated BB may have great potential to enlarge the adaptation in such disease. As an old drug, BB, a gift from the nature, still shines in the new era. However, the direct targeting molecule of BB was not elucidated and whether the BB's diabetic spermatogenesis rescuing effect in human as well remained to be further investigated.

## CONFLICT OF INTERESTS

All authors declared no conflict of interests.

## Supporting information

Supporting information

Supporting information

Supporting information

Supporting information
