# Supplementary material for: Berberine is sufficient to restore the destroyed seminiferous tubule structure and hypospermatogenesis in diabetes mellitus
Source: Clin Transl Med. 2020 Oct 11;10(6):e193. doi: 10.1002/ctm2.193 (PMC7548097; doi:10.1002/ctm2.193)
Supplement: Supplementary file 1 — Supporting information [file CTM2-10-e193-s001.docx]

**Table 1.**  Metabolic and physiological variables.

|  | **Con** | **DM** | | **DM+BB100** | **DM+BB200** | | **DM+BB400** |
| --- | --- | --- | --- | --- | --- | --- | --- |
| Initial weight (g) | 248.67±5.35 | | 254.17±10.32 | 249.17±7.39 | 250.00±9.57 | | 252.17±8.54 |
| Final weight (g) | 542.00±55.52 | | 226.5±30.83  ^a, **^ | 279.67±32.9  ^b, **^ | | 376.83±46.12  ^c, **^ | 409.17±28.95 ^de, **^ |
| Initial blood glucose (mmol/l) | 5.90±0.65 | 6.00±0.54 | | 5.58±0.48 | 6.01±0.0.75 | | 5.85±0.73 |
| Final blood glucose (mmol/l) | 6.38±0.65 | 18.37±3.29  ^a, **^ | | 19.13±5.56  ^b, **^ | 21.6±5.96  ^c, **^ | | 18.57±2.45  ^de, **^ |

† The results are shown as the mean ± standard. ^a, d^*P* compared with the Con group. ^b, c, e^*P* compared with the DM group.

**Legends**

**Supplementary Figure S1** Berberine attenuated the activation of ERK, p38 MAPK and JAK2 in the testis and Sertoli cells. (A) Representative Western blot results for ERK, p-ERK, p38, p-p38 in the TM4 cells of all five group (Con, HG, HG+10μM, HG+25μM, HG+100μM). (B-C) Expression of ZO-1, occludin in the TM cells from all five groups presented as bar graphs. (D) Representative immunohistochemistry results of JAK2 and p-JAK2 in testes from all five groups (x400). The arrows showed the localization of JAK2 and p-JAK2 in Sertoli cells. (E) Representative Western blot results for JAK2 and p-JAK2 in the testes of rats from all five groups. (F) Expression of JAK2 and p-JAK2 in five groups presented as bar graphs. (G) Representative Western blot results for JAK2 and p-JAK2 in Sertoli cells from all five groups. (H) Expression of JAK2 and p-JAK2 in five groups presented as bar graphs. Scale bars = 10 μm. n = 6 for each group. Data are expressed as mean ± standard deviation. ^a, b, d, f^*P* < 0.05 compared with the Con group. ^c, e, g^*P* < 0.05 compared with the DM group. ^a*, b*, d*, f*^*P* < 0.05 compared with the Con group. ^c*, e*, g*^*P* < 0.05 compared with the HG group. Con: Control; DM: diabetes mellitus; BB100: Berberine treat 100 mg/kg/d; BB200: Berberine treat 200 mg/kg/d; BB400: Berberine treat 400 mg/kg/d. HG: High glucose medium (50mM); 10μM,25μM,100μM: Berberine concentrations.

**Supplementary Figure S2.** AG490 and JAK2 siRNA improved the expression of ZO-1 and occludin by inhibiting JAK2/MAPK pathway in the Sertoli cells. (A) Representative Western blot results for ERK, p-ERK, p38, p-p38, ZO-1 and occludin in Sertoli cells from four groups (Con, HG, HG+BB, HG+AG490). (B) Phosphorylation of ERK and p38 in all four groups presented as bar graphs. (C) Expression of ZO-1 and occludin in all four groups presented as bar graphs. (D) Representative Western blot results for JAK2, ZO-1 and occludin in Sertoli cells from four groups (Con, HG, HG+vector, HG+siRNA). (E) Expression of JAK2, ZO-1 and occludin in all four groups presented as bar graphs. Data are expressed as mean±standard deviation. ^a, b, d^*P* < 0.05 compared with the Con group. ^c, e^*P* < 0.05 compared with the HG group. ^a*, b*^*P* < 0.05 compared with the Con group. ^c*^*P* < 0.05 compared with the HG group. Con: Control; HG: High glucose medium (45mM); BB: Berberine,50μM; AG490: A JAK2 inhibitor,50μM; Vector: siRNA vector; siRNA: siRNA that inhibits JAK2.

**Supplementary Figure S3.** Targeted inhibition of ERK and p38 pathway increased the expression of tight junction proteins. (A) Representative Western blot results for p38, p-p38, ERK, p-ERK, ZO-1 and occludin in Sertoli cells from five groups (Con, HG, HG+SB203580, HG+SCH772984, HG+SB203580+SCH772984). (B) Expression of JAK2, ZO-1 and occludin in all five groups presented as bar graphs. Data are expressed as mean±standard deviation. ^a^*P* < 0.05 compared with the Con group. ^b, c, d^*P* < 0.05 compared with the HG group. SB203580: A p38 inhibitor, 30μM; SCH772984: An ERK inhibitor, 300nM.
