# Supplementary figures and images for: Berberine is sufficient to restore the destroyed seminiferous tubule structure and hypospermatogenesis in diabetes mellitus
Source: Clin Transl Med. 2020 Oct 11;10(6):e193. doi: 10.1002/ctm2.193 (PMC7548097; doi:10.1002/ctm2.193)

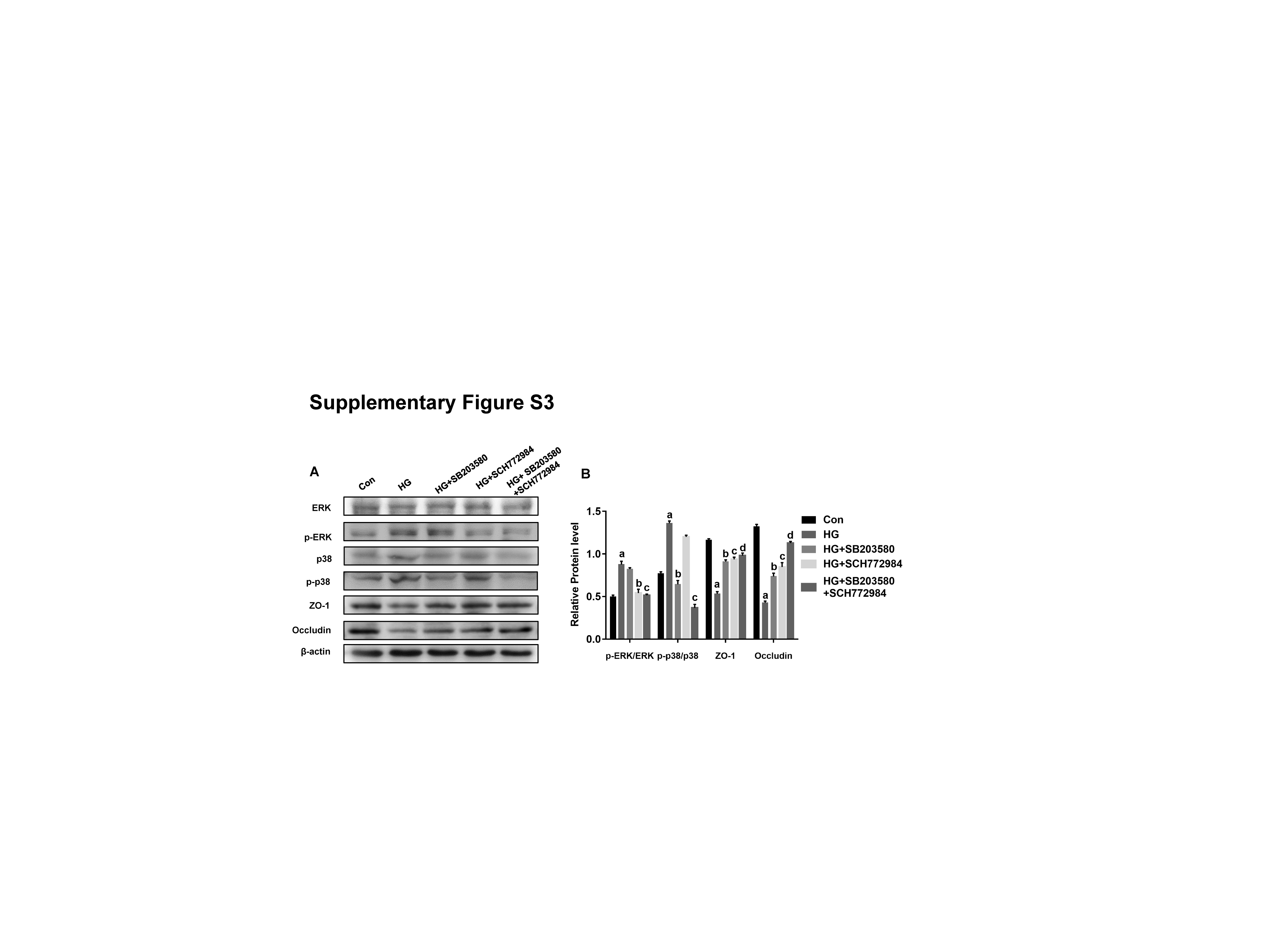

Supplement: Supplementary file 2 — Supporting information [file CTM2-10-e193-s002.tif]

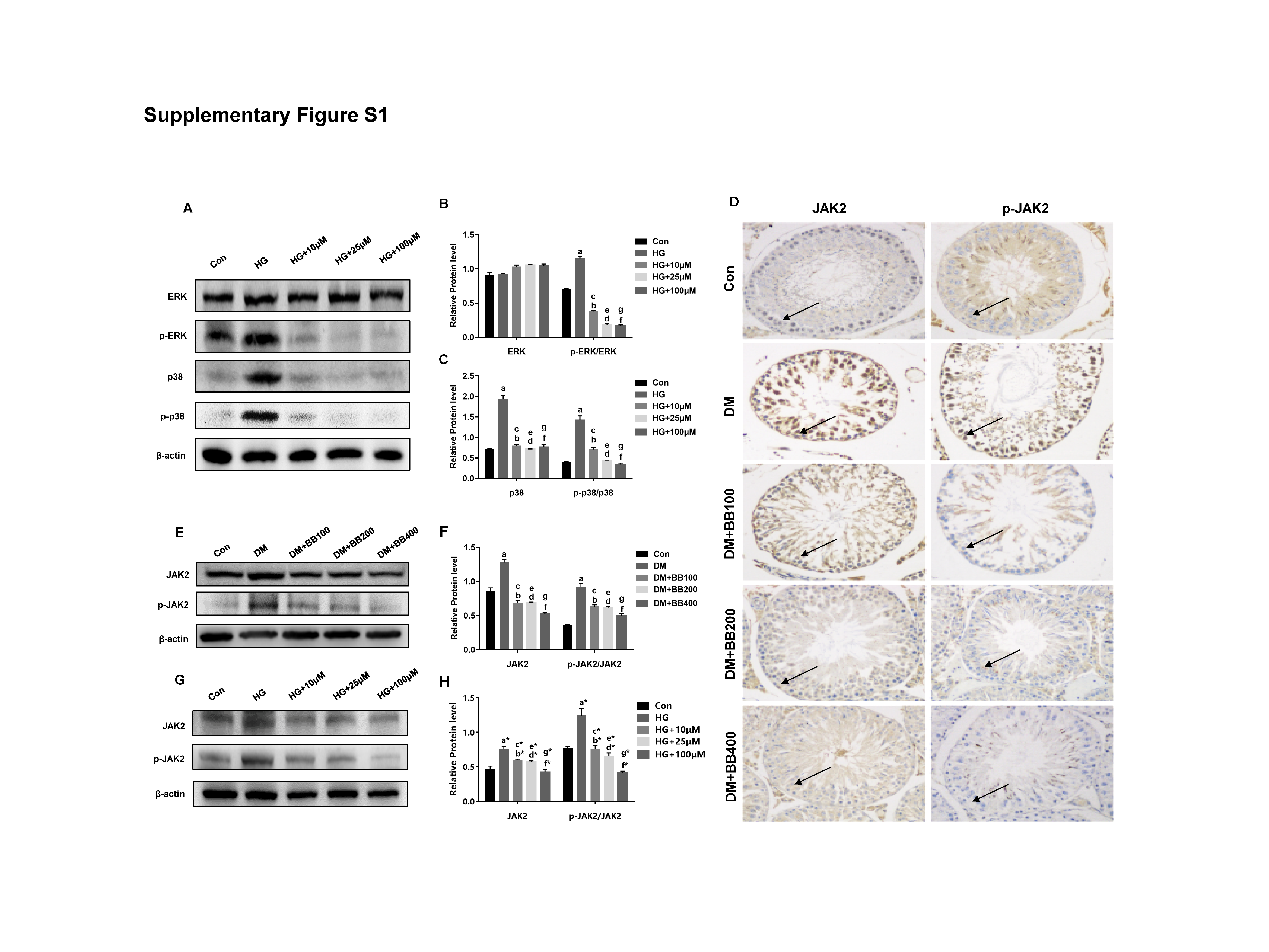

Supplement: Supplementary file 3 — Supporting information [file CTM2-10-e193-s003.tif]

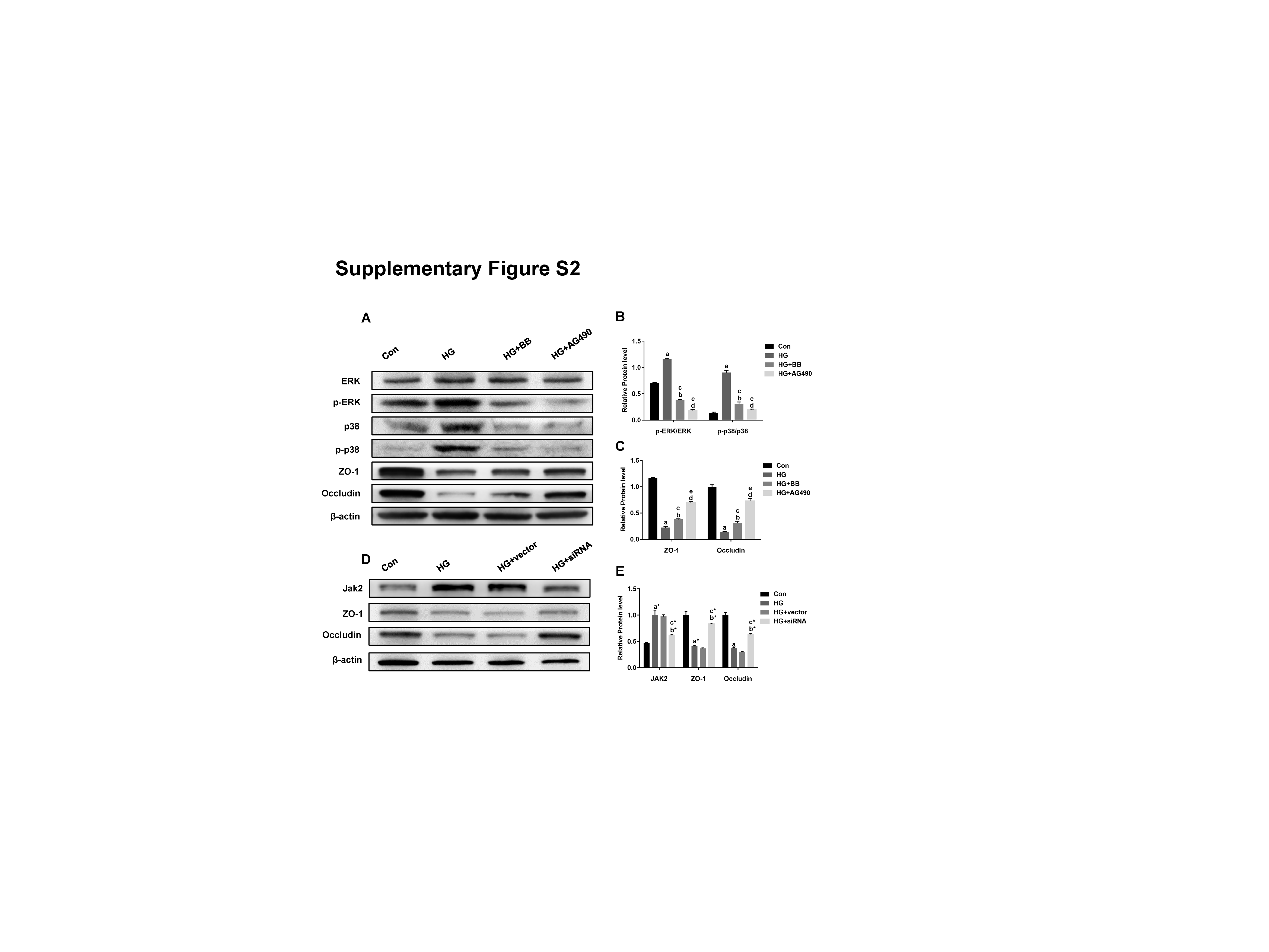

Supplement: Supplementary file 4 — Supporting information [file CTM2-10-e193-s004.tif]
